# A Pathway to Positive Youth Development: Unpacking the Asian American Youth Paradox and Cultural Orientations among Filipino American and Korean American Youth

**DOI:** 10.3390/children11080950

**Published:** 2024-08-06

**Authors:** Yoonsun Choi, Michael Park, Miwa Yasui

**Affiliations:** 1 Crown Family School of Social Work, Policy, and Practice, University of Chicago, Chicago, IL 60637, USA; myasui@uchicago.edu; 2 School of Social Work, Rutgers University, New Brunswick, NJ 08901, USA; p.michael@rutgers.edu

**Keywords:** Asian American youth, Filipino American youth, Korean American youth, internalizing problems, externalizing behaviors, academic performance, determinants of development

## Abstract

This study used longitudinal survey data of Filipino American and Korean American youth in the Chicago Metropolitan area (*N* = 786, *M_AGE_* = 15.00, *SD* = 1.91 at Wave 1 in 2014) to examine whether and how a set of organized predictors (i.e., universal predictors of youth outcomes and cultural orientations) independently and collectively explains internalizing and externalizing problems and academic performance. The results were that universal predictors such as youth antisocial beliefs, peer antisocial behaviors, and the quality of parent–child relations, were extensively predictive of youth outcomes in the expected directions. The magnitudes of universal predictors were largely unchanged when bilinear and multidimensional cultural orientation variables were accounted for together. The magnitudes of cultural orientation variables were slightly attenuated in full models but showed independent associations with youth outcomes. Specifically, English and heritage language proficiencies were protective of externalizing and internalizing problems. Behavioral practices in respective cultures increased youth problems. In addition, ethnic identity, although beneficial to mental health, can increase externalizing problems. The findings of this study provide insights into understanding the mixed outcomes among Asian Americans and important empirical evidence that can inform intervention programs to prevent youth problems, ultimately toward a pathway to positive youth development among Asian American youth.

## 1. Introduction

Positive youth development (PYD) encompasses a theoretical perspective or a program/policy approach that emphasizes strengths, resilience, and diversity in understanding youth developmental processes and aims to increase competency and autonomy among adolescents [1,2,3]. Despite racial stereotypes such as the model minority myth that portrays them as highly competent, efficacious, and self-sufficient, thus exemplary of PYD aims, Asian American youth in fact exhibit a mixed pattern of development, termed the “Asian American youth paradox” [4]. Compared to other races, Asian American youth as an aggregate report fewer externalizing problems and better grades, seemingly fitting the stereotype, but suffer from notably more internalizing problems. This pattern is unique because youth outcomes tend to share etiology and co-occur (e.g., [5,6]). The rates of internalizing problems among young Asian Americans are particularly alarming, with elevated rates of depression, anxiety, and suicidality [7,8,9]. Suicide remains the primary cause of death among young Asian Americans between 15 and 24 years old–the only racial group with this pattern [10,11]. It is deeply troubling that good academic performance and low externalizing problem behaviors can mask the high internalizing problems and mental health needs of Asian American youth [12], which leaves them unserved with serious and long-term consequences [13]. Discerning the etiology of the unique pattern bears scholarly and clinical importance and is a prerequisite to healthy development among Asian American children.

Predominantly children of first-generation immigrants, Asian American children and youth grow up in bicultural environments, e.g., families remain culturally Asian or Asian American while outside the home such as schools are dominated by Western White culture [14,15]. Bilinear cultural processes, i.e., acculturation of learning the dominant culture on the one hand and enculturation of maintaining the heritage culture on the other, take place in multiple dimensions (e.g., language, identity, and cultural practices) [16,17] and may complicate child development, particularly during the formative years of adolescence. Navigating their unique social positions as a visible racial and cultural minority, and further, as an immigrant family, Asian American youth face the compounding effects of minority and acculturative stresses [18]. Bilinear and multidimensional cultural orientations play a unique role in Asian American youth development and may be a key to understanding the mixed pattern of their development [4,19].

Only a few studies have empirically examined possible etiological sources of the paradox (e.g., [20]) and rarely focused on cultural orientations. Moreover, previous research efforts were often hampered by aggregating diverse Asian American subgroups and small sample sizes. Studies also tend to be specialized, i.e., examining one type of developmental outcomes or partial etiology, which likely restricts a comprehensive understanding. In addition, cultural orientation is hardly examined as a complex construct, despite its bilinear and multidimensional nature and the distinct roles of each component in youth development. Finally, longitudinal data is scarce for Asian Americans (and subgroup level is even scarcer) to contrast the concurrent and lasting impact of significant developmental predictors. Taking a holistic approach, this study included three distinct types of developmental outcomes and investigated whether they are similarly predicted by crucial clusters of predictors that include cultural orientations among Filipino American and Korean American youth samples, independently and collectively.

### 1.1. The Asian American Youth Paradox

Youth development is multifaceted—e.g., emotional, cognitive, and behavioral development, character and relationship building, academic behaviors as well as internalizing and externalizing problems, just to name a few. Nevertheless, they may share a common etiology, as there are distinct latent developmental clusters where positive developments in contrast to negative developments tend to cluster with a similar etiology [21,22,23]. For example, it is well known that internalizing problems (i.e., mental distress such as anxiety and depression, and social withdrawal), externalizing problems (i.e., negative behaviors including aggression, oppositionality, and property and status violations), and academic difficulties tend to co-occur in general adolescent populations [5,21,24]. In particular, adolescents who do well in school and do not engage in negative behaviors often report low rates of internalizing problems [5,24]. Likewise, high internalizing problems can be expressed through negative externalizing behaviors and poor academic performance [24,25]. Nevertheless, Asian American youth defy these patterns [4,20]. Furthermore, favorable outcomes seen at the aggregate level are not always observed at subgroup levels [26], and may not sustain during the transition to young adulthood, with internal struggles worsening over time [27]. Research findings on Asian Americans are frequently complicated by the diversity of culture, socioeconomic background, nativity, immigrant history and status, and geography among Asian Americans [28,29]. The complexity of Asian American youth development must be accounted for to the extent possible to accurately understand the multifaceted experiences and properly address their developmental needs.

### 1.2. Filipino American and Korean American Youth

Two Asian American subgroups, i.e., Filipino American and Korean American adolescents, were strategically chosen for their shared traits and crucial differences that can provide unusual opportunities to explain the Asian American youth paradox and the role of cultural orientations. For example, both groups immigrated to the U.S. mostly after the Immigration Act of 1965, and the families with adolescents in both groups are predominantly composed of first-generation parents and second-generation adolescent children who were either born in the U.S. or immigrated at an early age [28]. Filipino Americans and Korean Americans also are from similar socioeconomic status (SES), reducing a confounding class effect, but notably different in cultural orientation and racial experience [30]. For example, Filipino Americans are thought to be most assimilated among Asian American ethnic groups; they are mostly fluent in English, residentially integrated, and have started acculturation prior to immigration because of their colonial history [31,32]. Conversely, Korean Americans, especially first-generation immigrant adults, are closely bound to their culture, remaining predominantly monolingual, socializing primarily with those within their culture, and living in majority Korean neighborhoods [31]. These two groups also report distinct racial experiences due to their phenotypical differences, e.g., Filipino Americans are frequently mistaken for Hispanics or Latino/e/x [32,33].

Moreover, their adolescent children exhibit contrasting vulnerabilities, with a combination of commonality and differences in youth developmental outcomes, i.e., both groups of youth report mental health struggles, but vary in rates of antisocial behaviors and academic performance. For example, Korean Americans are “high achievers” in behavioral and educational outcomes [34] but contend with poor mental health [35], fitting the Asian American youth paradox. Conversely, Filipino American youth tend to report more problem behaviors [26] and higher rates of mental distress than other Asian subgroups [36], despite their higher SES advantage among parents. The points of overlap and divergence between Korean American and Filipino American adolescents are instrumental in underscoring the multivariate dimensions of the Asian American youth paradox.

### 1.3. Etiology of Asian American Youth Developmental Outcomes

The field of child and adolescent development has moved toward deconstructing existing Eurocentric foundations of human development. That is, the etiology of youth development including both risk and protective factors is comprised of universal factors (i.e., generalizable to youth of various races and ethnicities) and group-specific factors [20]. For example, several theoretical models of minority youth development that emerged during the recent decades (e.g., [37,38]) highlight the particular importance of racial positionality, heritage culture, and acculturation to accurately determine the developmental trajectory of racial/ethnic minority and immigrant youth. Existing research, while relatively sparse, suggests that the etiology of Asian American youth’s mixed outcome may be located in their unique social and cultural position [20]. In other words, added challenges from being a racial and ethnic minority and immigrant, such as being expected to develop cultural orientations, could be key to understanding the mixed pattern of youth development. However, it remains infrequent to examine universal factors and group-specific factors together to probe whether group-specific factors, such as cultural orientations, can meaningfully contribute to explaining youth development when universal factors, often dominant predictors, are considered simultaneously; and whether group-specific factors can indeed explain the idiosyncratic pattern of outcomes.

This study aims to fill these gaps by building a comprehensive model that accounts for several domains of etiology to explain Asian American youth development. Two integrative conceptual models, those of Garcia Coll et al. [37] and Mistry et al. [38], inform the construction of the model as they place a crucial emphasis on culture, race, and social positions. While accounting for several significant demographic control variables, this study organized etiological factors into two main clusters that may be most relevant to explain the developmental outcomes of Asian American youth. They are (1) universal predictors of youth outcomes and (2) bilinear and multidimensional cultural orientation variables. Each cluster is elaborated below.

#### 1.3.1. Universal Factors

Common examples of universal risk factors, in particular for youth delinquency, are antisocial beliefs of self and antisocial peers [39]. When children believe it is okay to commit antisocial behaviors (such as fights and theft) or have friends whose behaviors are antisocial, they may be inclined to engage in negative behaviors. In addition, numerous family process indicators continue to serve as strong predictors of youth development even though peer influences become notable and powerful during adolescence. Universal aspects of parenting, including lack of parental monitoring and rules [40,41], low parental affection/warmth [41,42], weak parent-child bonding, and high parent-child conflict [43], are extensively shown to predict internalizing problems [41], externalizing problems [41,43], and poor academic performance [42] across cultures and ethnicities. In the present study, we examined these parental dimensions as part of the universal predictors that are likely to influence Asian American youth in addition to antisocial beliefs and peer behaviors. In addition, explicit parental affection was included in this study. Explicit expression of parental affection and warmth (e.g., verbal and physical expression of parental love) is often thought to be more Western but in contrast to implicit expression that is more common among Asian and Asian American families and beneficial to youth development [44], explicit expression is a significant and stronger predictor of positive youth development among Asian Americans [20] and was included as one of the universal predictors in this study.

#### 1.3.2. Cultural Orientations

As a racial/ethnic and cultural minority, cultural orientation is one of the significant issues facing Asian American families [45]. After years of scholarly debates, cultural orientation is now commonly conceptualized as bilinear [i.e., including both *acculturation* (cultural orientation to the mainstream, learning and adopting the host culture), and *enculturation* (or cultural orientation to the culture of origin, preserving one’s heritage culture)] and multidimensional (i.e., encompassing multiple dimensions such as language, identity, values, and behaviors) [17]. Recent empirical studies show that each component contributes uniquely to the family process and youth development [46,47]. Accordingly, this study includes six cultural orientation variables that reflect both bilinearity and multidimensionality. That is, heritage and English language proficiency, heritage and host culture practice, and American and ethnic identity. Ethnic identity [48], participation in both heritage and American culture [49], and bilingualism [50] have been generally found to be protective factors, with some mixed findings. In addition, as Choi et al. [16] explicated, biculturalism may have differential implications across Asian American subgroups [51].

### 1.4. Present Study

Only a handful of studies have empirically examined the unique etiology of Asian American adolescents. To address this gap, we constructed a comprehensive model that accounts for both universal and group-specific factors that impact Asian American youth development. Specifically, we created two broad clusters of etiological factors: (1) universal factors of youth outcomes, that include youth antisocial beliefs, antisocial peers, parent-child bonding and conflict, explicit parental affection, parental rules and monitoring, and (2) cultural orientation factors, including host and heritage culture language proficiency, host and heritage culture practice, and American and ethnic identity. We investigated how each cluster predicts externalizing problems (i.e., substance uses and antisocial behaviors), academic performance (i.e., GPA), and internalizing problems (i.e., depressive symptoms). A central goal of this study was to determine the independent and collective effects of universal and culture-specific factors on Asian American youth development, possibly the Asian American youth paradox. We examined the predictive relationships concurrently and longitudinally. Previous developmental outcomes are one of the powerful predictors of later outcomes [52]. Thus, we ran an alternate set of longitudinal models that included the outcome from the previous wave. Ethnic-specific effects for these associations were also examined (Filipino Americans vs. Korean Americans).

The primary focus of this study is to establish a conceptual model that is more expansive than those in many existing empirical studies to better account for the unique social positionality of Asian Americans and the study hypotheses were generated both from the literature on samples undifferentiated by race or exclusive of Asian Americans and the limited literature on Asian Americans. For example, we expected universal factors to predict youth outcomes based on the general trends as previously described, independent of the cultural orientation factors. That is, antisocial beliefs, peer antisocial behaviors, parent-child conflict would be harmful, while parent-child bonding, explicit affection, and rules and monitoring would be beneficial to youth development. We also expected that findings for cultural orientation would parallel those in prior studies. That is, we hypothesized that ethnic identity, participation in both heritage and American cultures, and bilingualism would decrease mental distress and externalizing behaviors and increase GPA. Existing literature is limited in delineating ethnic group differences across Filipino American and Korean American youth in these associations and studies that do exist focus on differences in characteristics of cultural orientation across Asian American subgroups but not in how cultural orientation is linked with youth outcomes. Accordingly, we did not expect interactions by ethnicity to be significant. We explored possible moderations in case either Filipino Americans or Korean Americans show particular vulnerability to inform future investigations.

It should be noted that a similar approach was used to examine unique contributions of Asian American family processes as group-specific factors (e.g., gendered norms, academically oriented parental control, family-centered activities and implicit affection), while accounting for universal factors (i.e., [20]). The readers may find similarities in some of the variables, analytic approaches and other sections with the Section 2.

## 2. Materials and Methods

### 2.1. Overview of the Project

This study used data from the Midwest Longitudinal Study of Asian American Families (MLSAAF) that started in 2014 to follow Filipino American (FA) and Korean American (KA) families in the Chicago Metropolitan areas. The MLSAAF is ongoing and recently launched its Wave 5 (W5) of the longitudinal data collection. At the baseline, eligibility criteria were as follows: families whose mothers are of Filipino and Korean heritage (self-identification) with children aged 12 to 17, residing in Chicago or surrounding 4 major counties. Recruitment of participants involved multiple sources, including phonebooks, schools (both public and private), ethnic religious institutions such as Protestant and Catholic churches, and Buddhist temples. Outreach to the targeted communities were intentionally and systematically conducted until the project reached its goal of 350 families per group. The total sample at W1 was 1574 from 804 families (389 FA and 415 KA). Data for W2 was collected later in 2016, with a 79% retention rate. Starting W3, a significant proportion of youth participants became emerging adults. Thus, this study used waves 1 and 2 when the child participants were mostly adolescents. There was no significant difference in demographics between those who participated in only one of the waves or both waves. Data collection in W1 was an in-person, interviewer-assisted method, and a self-administered survey in later waves. The self-administered survey was available in paper and online and in three languages: English, Korean, and Tagalog. Only 8% of youth participants (61 KA youth and 2 FA youth) completed the survey in their heritage language. We excluded two Tagalog surveys from the analysis because they, unusual among Filipino American youth, may have markedly different cultural orientations from others who used English surveys.

### 2.2. Baseline Sample Characteristics

FA and KA youth were roughly equal in the number of males and females. Youth average age was 15.27 (*SD* = 1.88) for FA and 14.76 (*SD* = 1.91) for KA. The sample included U.S.-born (71% FA, 58% KA) and those who immigrated at a young age. Parents’ average age was 46.13 (*SD* = 5.75) for FA and 45.28 (*SD* = 3.77) for KA, and were predominantly foreign-born (91% FA, 99% KA), married (89% FA, 93% KA), well-educated (94% FA, 86% KA having some college education). These demographics, i.e., highly educated middle-income families, parallel characteristics of FA and KA families observed in Census or nationally representative data such as Add Health.

### 2.3. Measures

Unless noted otherwise, responses for all measures were provided using an ordinal Likert scale, that ranged from 1 to 5, e.g., (1) not at all or never to (5) strongly or always.

### 2.4. Independent Variables

#### 2.4.1. Universal Factors

To assess universal predictors of youth development, the following measures were used: (1) *Youth Antisocial Beliefs* used 11 items from the Seattle Social Development Project [53] that asked youth whether it is okay for someone at their age to, e.g., have sex, get drunk, and carry a gun/knife. (α = 0.86 for FA; 0.88 for KA); (2) *Peer Antisocial Behaviors* consisted of 7 items from the Raising Healthy Children Project [54] about close friends and their antisocial behaviors, e.g., how many of their ten closest friends have done negative behaviors or used substance. (α = 0.73 for FA; 0.66 for KA). Response options for this scale were (1) None to (5) Most of them (9–10 friends); (3) *Parent-Child Conflict* included 4 questions about how often parent and child argue or fight or get angry at each other [55] (α = 0.83 for FA; 0.79 for KA); (4) *Parent-Child Bonding* used 5 questions from Add Health, that asked how much the child feels close to their mom. (α = 0.88 for FA; 0.85 for KA); (5) *Explicit Affection*, consisted of 9 items from Parental Acceptance and Rejection Questionnaires [56]. Examples for this scale are “My mom says nice things about me.” (α = 0.90 for FA; 0.90 for KA); (6) *Rules* consisted of a range of rules that parents may enforce including curfew, house chores, and rules around school work. A total score was calculated by counting the number of parental rules, ranging from 0–6; (7) *Monitoring* was a 1 item that asked how often parent knows where their child is.

#### 2.4.2. Cultural Orientations

Six scales assessed bilinear and multidimensional cultural orientations. (1) *Language proficiency* was assessed using the Language, Identity, and Behavior (LIB) scale [57], which included a total of four (two sets of two parallel items) that measured youth language proficiency in speaking and understanding either the host language (English) or heritage language (Filipino or Korean). [*r* = 0.60 (FA) and 0.73 (KA) for English; *r* = 0.73 (FA) and 0.81 (KA) for heritage language]. (2) *Cultural Practice* was also taken from the LIB [57] 10 items asking about participation in either American or heritage cultures such as social gatherings, media use, and racial makeup of peers [α = 0.75 (FA) and 0.75 (KA) for host cultural participation; α = 0.79 (FA) and 0.74 (KA) for heritage cultural participation]; (3) *Identity* also used 10 questions from LIB [57], that asked how much youth identified themselves as American or Filipino/Korean [α = 0.81(FA) and 0.77 (KA) for *American Identity*; α = 0.76 (FA) and 0.77 (KA) for *Ethnic Identity*].

#### 2.4.3. Control Variables

Several demographic variables were included as controls. For example, family SES is one of the most powerful predictors of youth development [58] and was assessed as self-perceived family SES (1 lower to 5 upper class). Older age predicts more problems and youth age was included. In addition, internalizing and externalizing problems and academic achievement vary across nativity and gender. Specifically, foreign-born Asian American adolescents were found to have lower rates of mood/anxiety disorders and lower problem behaviors than U.S.-born counterparts [59]. Nativity was assessed by a place of birth (0 foreign, 1 U.S.-born). Girls generally report higher internalizing problems than boys who tend to report more externalizing problems [40]. Gender was coded as 0 girls, 1 boys. Youth ethnicity (0 FA, 1 KA) was included to account for group mean/proportion differences in the outcomes and to enable interaction terms in later models.

### 2.5. Dependent Variables

#### 2.5.1. Substance Use

Substance use behavior was assessed with the following items, e.g., having ever smoked, drank, drank without adults present, or used marijuana, and cocaine/crack. Nearly all participants reported no use, resulting in a heavily skewed distribution of rates of substance use. As a result, we created a binary outcome variable where 1 = use of any drugs and 0 = no substance use.

#### 2.5.2. Antisocial Behaviors

Antisocial behavior was measured using a total of nineteen antisocial behaviors during the past year prior to the survey (e.g., bullying, getting into fights, using a weapon, stealing, or skipping school without an excuse). Participants responded with either a no (0) or yes (1) for each item. Positive responses were summed as a count variable that ranged from 0 to 19.

#### 2.5.3. GPA

Academic performance was assessed by GPA, which was calculated by using youth’s most recent grades in English, math, social studies, and science. The scale ranged from 1 to 4.

#### 2.5.4. Depressive Symptoms

Internalizing problems were assessed by 13 items from the Children’s Depressive Inventory [60] that asked about depressive symptoms during 2 weeks prior to the survey, e.g., not enjoying anything at all, feeling lonely (α = 0.93 for FA; 0.92 for KA).

### 2.6. Plan of Analysis

Using STATA v 15.1, multiple hierarchical regression models were estimated. For binary outcomes such as substance use, logistic regression was employed. Negative binomial regression was used for count outcomes such as antisocial behaviors, and the Ordinary Least Squares (OLS) regression was applied to continuous outcomes, i.e., GPA and depressive symptoms. Various methods can be used to estimate a count variable outcome. Following Swartout, et al. [61], several methods including Poisson, negative binomial, and zero-inflated Poisson models were compared using fit indices including AIC and BIC, and it was determined that negative binomial regression provided the best model fit.

#### 2.6.1. Universal Factors

In the first set of Models 1(a), a cluster of universal factors (i.e., youth antisocial beliefs, antisocial peers, parent-child bonding and conflict, explicit parental affection, parental rules, and monitoring) was added with five demographic controls (ethnicity, youth’s place of birth, family SES, age, and gender) to examine whether and how it explains, respectively, four individual youth outcomes (i.e., substance use, antisocial behaviors, GPA and depressive symptoms), controlling for demographics.

#### 2.6.2. Cultural Orientation Factors

Similar to the above, in Models 1(b), a cluster of cultural orientation factors (i.e., host and heritage culture language proficiency, host and heritage culture practice, and American and ethnic identity) was regressed along with five demographic controls on four youth outcomes.

#### 2.6.3. Comprehensive Model

Models 2 were a comprehensive model in which demographics and two clusters were regressed together to predict four youth outcomes individually.

#### 2.6.4. Interaction Terms

In Models 3 that examined whether these relationships vary by ethnicity, interaction terms (each predictor × ethnicity) were added to Model 2. Each cluster was added to Model 2 one at a time, resulting in Model 3(a) and 3(b), to examine whether the relation between predictors in each cluster and youth outcome varies by ethnicity, while accounting for other cluster of predictors and controls.

#### 2.6.5. Concurrent vs. Longitudinal Models

To test both concurrent and longitudinal relationships between predictors and youth outcomes, with an exception of substance use that was not collected in W2, we used youth outcomes from both W1 and W2. In all models, we first ran concurrent models with both predictors and outcomes from W1, then longitudinal models with predictors from W1 and outcomes from W2 to examine the relationships over an approximately 1.5-year timeframe. Finally, we examined another set of longitudinal models that included the same outcome from the previous year as one of the predictors.

#### 2.6.6. Additional Analysis Notes

Continuous variables were standardized to facilitate the interpretation of the interaction terms. The likelihood ratio (LR) test was conducted to assess significant fit increments in hierarchical regression models. When the LR test between Model 2 (only with main effects) and each of Model 3(a) and 3(b) was significant, the slopes of the interaction terms were plotted graphically to show the relationships. We did not find multicollinearity among variables (i.e., 5 > the variance inflation factors (VIF)). No missing data imputation was necessary because the missing rates in variables of both W1 and W2 were less than 5% [62]. To estimate the effect sizes of each predictor, we calculated the odds ratio (*OR*) for logistic regression and the partial eta-squared (η_p_^2^) for linear regression models.

## 3. Results

In Table S1, we provide a summary of descriptive statistics of the study variables including proportions and means of outcome variables and bivariate correlations for the full sample. Table S2 provides bivariate correlations by ethnic groups. The patterns of descriptive statistics were largely as expected. In hierarchical regression models (summarized in Tables S3–S6 for each outcome variable), there was no notable difference in the regression coefficients of predictors and statistical significance from Models 1(a) and 1(b) to Model 2. Thus, the results of a comprehensive final model (Models 2) are mainly discussed in the text. Because interaction terms were mostly non-significant in Models 3(a) and 3(b), we did not provide the results in tables. Two statistically significant interaction coefficients are plotted in Figures S1 and S2.

### 3.1. Universal Factors

Universal factors in Model 1(a) were extensively significant predictors of all types of youth outcomes. Magnitudes and significance of coefficients in Model 1(a) generally remained the same in Model 2 when cultural orientation variables were added. *Youth Antisocial Beliefs* concurrently predicted higher substance use (*b* = 0.72 [*OR* = 2.06], *p* < 0.001) and higher antisocial behaviors (*b* = 0.33 [*OR* = 1.39], *p* < 0.001) and concurrently (*ß* = 0.10, *p* < 0.001, η_p_^2^ = 0.016) and longitudinally (*ß* = 0.09, *p* < 0.05, η_p_^2^ = 0.010) predicted more depressive symptoms. Contrary to the expectation, it did not predict GPA. *Peer Antisocial Behaviors* also significantly and extensively predicted externalizing behaviors. It concurrently predicted more substance use (*b* = 0.76 [*OR* = 2.13], *p* < 0.001). Most notably, it predicted concurrently (*b* = 0.35 [*OR* = 1.42], *p* < 0.001) and longitudinally (*b* = 0.28 [*OR* = 1.32], *p* < 0.01) higher antisocial behaviors and remained marginally significant when prior antisocial behaviors were adjusted for (*b* = 0.15 [*OR* = 1.17], *p* < 0.10). It also concurrently (*ß* = −0.07, *p* < 0.001, η_p_^2^ = 0.017) and longitudinally (*ß* = −0.05, *p* < 0.05, η_p_^2^ = 0.008) predicted lower GPA. It, however, did not predict internalizing problems, i.e., depressive symptoms. *Parent-Child Conflict* and *Parent-Child Bonding* were significantly associated in the expected directions with antisocial behaviors and depressive symptoms. Specifically, *Parent-Child Conflict* was linked with higher antisocial behaviors concurrently (*b* = 0.20 [*OR* = 1.23], *p* < 0.01) and longitudinally in Model 1(a) (*b* = 0.19 [*OR* = 1.20], *p* < 0.05) but the significance level changed to *b*= 0.15 [*OR* = 1.17], *p*< 0.10 in Model 2. It also predicted more depressive symptoms concurrently (*ß* = 0.17, *p* < 0.001, η_p_^2^ = 0.042) and longitudinally (*ß* = 0.11, *p* < 0.01, η_p_^2^ = 0.012) Conversely, *Parent-Child Bonding* predicted less antisocial behaviors concurrently (*b* = −0.19 [*OR* = 0.83], *p* < 0.01) and longitudinally (*b* = −0.27 [*OR* = 0.77], *p* < 0.05) and concurrently fewer depressive symptoms (*ß* = −0.10, *p* < 0.01, η_p_^2^ = 0.010). *Explicit Affection* longitudinally predicted fewer depressive symptoms in Model 1(a) (*ß* = −0.11, *p* < 0.05, η_p_^2^ = 0.009) and marginally in Model 2 (*ß* = −0.09, *p* < 0.10, η_p_^2^ = 0.006) but was not associated with other types of youth outcomes. *Rules* concurrently predicted associated with antisocial behaviors (*b* = 0.10 [*OR* = 1.11], *p* < 0.10) and GPA (*ß* = −0.04, *p* < 0.10, η_p_^2^ = 0.005) at marginal significance but in the opposite direction, i.e., more antisocial behaviors and lower GPA. *Monitoring* concurrently predicted less antisocial behaviors (*b* = −0.14 [*OR* = 0.87], *p* < 0.01) and higher GPA (*ß* = 0.04, *p* < 0.05, η_p_^2^ = 0.006) but more depressive symptoms (*ß* = 0.09, *p* < 0.01, η_p_^2^ = 0.015) and longitudinally predicted higher GPA (*ß* = 0.05, *p* < 0.05, η_p_^2^ = 0.008). It did not predict substance use.

### 3.2. Cultural Orientations

English and heritage language proficiency were respectively and significantly associated with all youth outcomes, except substance use in which no relation was found. These associations were significant either concurrently or longitudinally or both. Specifically, *English Language* concurrently predicted less antisocial behaviors (*b* = −0.15 [*OR* = 0.86], *p* < 0.01), better GPA (*ß* = 0.08, *p* < 0.001, η_p_^2^ = 0.021) and less depressive symptoms (*ß* = −0.08, *p* < 0.01, η_p_^2^ = 0.010). *Heritage Language* was both concurrently (*b* = −0.15 [*OR* = 0.86], *p* < 0.05) and longitudinally (*b* = −0.19 [*OR* = 0.82], *p* < 0.10) associated with less antisocial behaviors and longitudinally fewer depressive symptoms (*ß* = −0.12, *p* < 0.01, η_p_^2^ = 0.012) and this longitudinal relation remained when prior level of depressive symptoms was accounted for (*ß* = −0.10, *p* < 0.05, η_p_^2^ = 0.010). *Heritage Language* also concurrently predicted higher GPA at marginal level (*ß* = 0.05, *p* < 0.10, η_p_^2^ = 0.005). Both *Host Culture Practice* and *Heritage Culture Practice* longitudinally predicted more antisocial behaviors (*b* = 0.19 [*OR* = 1.21] and *b* = 0.25 [*OR* = 1.28] respectively, *p* < 0.05), which remained significant (*b* = 0.21 [*OR* = 1.24] and *b* = 22 [*OR* = 1.25] respectively, *p* < 0.05) when prior antisocial behaviors were accounted for. Regarding GPA, *Host Culture Practice* longitudinally predicted a higher GPA (*ß* = 0.04, *p* < 0.10, η_p_^2^ = 0.005), whereas *Heritage Culture Practice* predicted a lower GPA (*ß* = −0.05, *p* < 0.10, η_p_^2^ = 0.006) both at marginal level but became significant at *p* < 0.05 when a prior GPA was accounted for (*ß* = 0.05 and −0.06, *p* < 0.05, η_p_^2^ = 0.009 and 0.012). Further, *Heritage Culture Practice* also predicted more depressive symptoms concurrently (*ß* = 0.10, *p* < 0.01, η_p_^2^ = 0.014) and longitudinally (*ß* = 0.07, *p* < 0.10, η_p_^2^ = 0.005). *American Identity* was concurrently associated with less substance use in Model 1(b) (*b* = 0.27 [*OR* = 0.77], *p* < 0.05) but became nonsignificant in Model 2. *American Identity* predicted a lower GPA when a prior GPA was accounted for (*ß* = −0.05, *p* < 0.05, η_p_^2^ = 0.008). *Ethnic Identity* too concurrently predicted a lower GPA (*ß* = −0.04, *p* < 0.05, η_p_^2^ = 0.006). Both identities, however, concurrently predicted fewer depressive symptoms (*ß* = −0.06, *p* < 0.10, η_p_^2^ = 0.005 for *American Identity ß* = −0.06, *p* < 0.05, η_p_^2^ = 0.005 for *Ethnic Identity*).

### 3.3. Interactions by Ethnicity

Following the LR test between Model 2 and each of Model 3(a) and 3(b), we found two interaction terms (*Peer Antisocial Behaviors* × *Ethnicity* [*ß* = 0.13, *p* < 0.05, η_p_^2^ = 0.007]; *Heritage Cultural Practice* × *Ethnicity* [*ß* = −0.13, *p* < 0.05, η_p_^2^ = 0.006] to be statistically significant at *p* < 0.05 level (See Figures S1 and S2). First, the main effect of *Peer Antisocial Behaviors* on depressive symptoms was not statistically significant concurrently but was significant only among KA youth (*b* = 0.10, *p* < 0.05) (Figure S1). The concurrent effect of *Heritage Culture Practice* on depressive symptoms was only significant among FA youth (*b* = 0.17, *p* < 0.001) (Figure S2).

## 4. Discussion

With an expansive approach, this study empirically examined how distinct clusters of predictors pertinent to Asian American youth may explain, respectively and collectively, youth outcomes and possibly contribute to their mixed pattern of outcomes. The findings of this study offer important empirical evidence that can meaningfully advance our knowledge of Asian American youth development. For example, the study found that each cluster of predictors was mostly independent of one another in explaining youth outcomes, showing their unique contributions. The changes largely in coefficient magnitudes but not in statistical significance in the final full models further indicate that predictors in a cluster are modestly correlated with predictors in other clusters (as indicated by low VIF) and that there may be modest mediation between clusters. For example, a child’s strong ethnic identity may help build parent-child bonding among Asian American families, suggesting a partial-mediating pathway [46], but each variable is a uniquely significant predictor of youth adjustment. In addition, the significant associations found in the final models of this study are particularly reassuring since another important cluster of variables was simultaneously accounted for.

Specifically on each cluster, first, the results showed the salience of universal predictors, which confirms a commonality of the child’s core developmental process regardless of racial/ethnic background. In addition, while universal predictors remain significant and robust in the comprehensive model, cultural orientation variables, as bilinear and multidimensional constructs, were independent and statistically significant predictors of Asian American youth development, showing their distinct and dimension-specific impact on youth development. Importantly, the findings also show how internalizing vs. externalizing problems and academic performance, while sharing several common predictors, are determined by different sets of predictors and the complexity of findings (e.g., mixed effects of some predictors) offers some insights to understand the Asian American youth paradox. These results were mostly similar across Filipino American and Korean American youth, confirming the shared developmental processes in how each predictor influences youth development across Asian American subgroups. Each point is elaborated in the following along with implications for practice and intervention when relevant.

### 4.1. Universal Predictors

This study confirmed that universal predictors were strong and extensive predictors of all types of youth outcomes and equally so across Filipino American and Korean American youth. Consistent with existing studies that often rely on White samples or samples exclusive of Asian Americans, we found that youth antisocial beliefs, antisocial peer behaviors, parent-child relationships (conflict, bonding), and parenting behaviors (explicit affection, rules and monitoring) were powerful determinants of youth outcomes among Filipino American and Korean American youth and that their impact remained robust when an array of cultural orientation variables were accounted for. Universal factors are often sidestepped as a research focus on Asian Americans, perhaps because relatively lower rates of externalizing problems among them do not call for attention [63]. Our findings highlight the notable significance of universal processes that youth from any cultural and racial background including Asian American youth are equally vulnerable to, such as peer influences and family dynamics, on a range of developmental outcomes.

The study also found several significant and differential impacts on different types of developmental outcomes. For example, youth antisocial beliefs predicted all adjustment outcomes but not academic performance. Our data cannot confirm whether this finding is unique to Asian Americans and explains the Asian American paradox because we did not have other comparative groups. Additional comparative investigations are warranted. Conversely, peer behaviors were influential in externalizing problems and academic performance but, not mental health. This finding confirms the existing peer literature, for example, that peer pressures can elicit behaviors that youth normally may not do without them [64,65]. Although mental distress can be expressed in the form of externalizing problems, thus they are related to one another, peer influences appear mainly on external behaviors that peers can put pressure on, but not on the internal, mental state. It should not be overlooked, however, that mental health among Korean American youth was adversely influenced by peer antisocial behaviors. Explicit affection from parents was particularly beneficial to mental health, underscoring the importance of practicing explicit affection in Asian American families. In addition, as expected, parent-child bonding was essential in youth adjustments. Taken together, Asian American families should be assisted to strengthen their relational bonding, e.g., via practicing more explicit affection. The focus on improving explicit affection is especially justified as Asian parents struggle to express their love and affection verbally and physically [66]. While implicit affection is also beneficial, especially for academic outcomes [20], the importance of explicit affection needs to be emphasized to reduce mental distress among Asian American youth especially given the serious rate of mental distress among the group.

Practicing various types of parental monitoring or controlling behaviors, which are supposedly prevalent and relatively higher among Asian American families, may explain the mixed outcomes of Asian Americans as they have variant impacts on youth adjustments. For example, parental monitoring was helpful for externalizing problem behaviors and academic performance but increased mental distress, which may explain the Asian American youth paradox. This study used a single-item measure of parental monitoring, i.e., parents knowing their child’s whereabouts. Parental monitoring can limit opportunities for risky behaviors, thus helpful for externalizing behaviors. However, parents’ knowing where their child is what the child shares, therefore reflects the quality and dynamics of parent-child relationships [67]. Therefore, it is intriguing that it nonetheless is associated with higher mental distress among this study group. Interestingly, another study using the MLSAAF data found that Asian American parental control specific to academic performance, called “academic control”, may increase mental distress and may not help academic performance, the very outcome that such control aims to improve. Academic control includes behaviors, such as making sure youth do homework and not allowing social activities so that youth can do schoolwork. Furthermore, parental rules predicted outcomes in the opposite direction. While it is true that several family variables were modeled together which could produce suppression effects in multivariate regression models, parental rules and parent-child conflict were positively correlated in bivariate relations, suggesting that parental rules during adolescence are likely a source of conflict and may not help youth development. We do not mean to say that parental rules are flat-out harmful to youth development since there is abundant research that documents the important role of parental rules in helping regulate youth behaviors (e.g., [67]). However, given the findings on parental monitoring and rules of this study and others, additional investigation is warranted to identify appropriate levels and kinds of parental rules and monitoring and parental control that can be helpful for Asian American adolescents. For example, granting autonomy during adolescence is consistently shown to be beneficial [68]. A well-balanced set of rules, monitoring, and autonomy is likely most helpful to positive youth development among Filipino American and Korean American youth.

Particularly relevant to Asian American families, it was notable that the magnitude of parent-child conflict decreased when cultural orientations were added. The source of parent-child conflict is often derived from acculturation differences between parents and children in Asian immigrant and Asian American families. Indeed, the MLSAAF data has shown that the increase in mental distress from adolescence to young adulthood between 2014 (Wave 1) and 2018 (Wave 3) was partly due to increases in intergenerational cultural conflict and acculturative stress [69]. The finding of this study suggests that cultural orientations, especially acculturation discrepancy between parents and children, should be a target of intervention to mitigate mental distress among Asian American youth. A study on Vietnamese and Cambodian youth shows that intergenerational cultural gaps per se may not predict youth maladjustments and they become an issue only if they lead to parent-child conflict [70]. Interventions could be designed to help families understand and accept acculturation differences in the family and to prevent escalating to actual parent-child conflict. Moreover, parent-child conflict, a significant predictor of negative outcomes, especially mental health, may explain the Asian American paradox. In other words, immigrant families are known to be highly motivated and driven to social mobility, which may explain the low rate of externalizing problems and high academic performance. However, it is also true that growing up in an immigrant family where intergenerational cultural conflict is normative may contribute to a high level of mental distress because of subsequent parent-child conflict.

### 4.2. Cultural Orientations

In this study, cultural orientations showed a mixed set of results. As bilinear and multidimensional constructs, different dimensions of cultural orientation were associated differently with specific youth outcomes. First, language proficiency in either host or heritage language, collectively bilingualism, was protective. The benefit of language proficiency, in particular bilingualism and retention of heritage language, has been extensively reported [50] and this study provides additional empirical findings. The benefits were extensive, i.e., across internalizing and externalizing youth outcomes as well as academic performance, and remained statistically significant after universal factors were accounted for. Heritage language proficiency notably showed a lasting impact on reducing externalizing problem behaviors and improving mental health. With this extensive support for bilingual competence, Asian American families should be encouraged to strengthen bilingual competence and maintain their heritage language. The heritage language retention among U.S.-born Asian Americans can be challenging with Asian language classes not frequently offered. However, we have seen increases in Chinese and Korean class offerings in recent years, and given its noticeable impact on youth adjustments and positive development, formal and informal venues to foster bilingualism and the retention of heritage language should be promoted.

Cultural practices in both host and heritage cultures in the final models were not protective in externalizing problem behaviors and, among Filipino Americans, mental health. The impact on academic performance was also mixed. That is, host cultural practices were predictive of a higher GPA when the opposite was the case for heritage cultural practices. Moreover, in bivariate associations, although largely nonsignificant, any significant correlations were positive relations with negative outcomes. In a previous study with Korean American early adolescents [46], a negative association between host culture practice and youth outcomes (both antisocial behaviors and depressive symptoms) was reported, and similar to this study, other dimensions of cultural orientations (e.g., identity and language) were modeled together. In another study with Korean American adolescents, heritage culture practices too were associated with depressive symptoms [71]. This study extends that heritage culture practices may also increase externalizing problem behaviors and decrease academic performance. It is not clear why cultural practices in either culture emerge generally as risk factors. Given that the items for this dimension ask about hanging out with friends, media consumption, and social gathering, when other dimensions of cultural orientations such as identity and language are modeled together, these items may be prone to be associated with more problems. Additional probe should be made to better understand this finding.

Finally, the findings on both American and ethnic identity were also complicated as they predicted lower GPA concurrently or longitudinally but less mental distress. American identity in particular showed a lasting negative impact on GPA even after accounting for a prior GPA. At the same time, considering its bivariate relation to the outcomes, its positive role in reducing mental health issues seemed notable. An earlier study with a diverse racial/ethnic group of youth showed that a stronger ethnic/racial identity could increase externalizing problem behaviors [72], which this study did not find. We instead found that American identity was associated with lower substance use. Overall, these findings illustrate the complexity of cultural orientations, especially identity development, and its role in shaping youth adjustments, echoing the findings of Yip and her colleagues [73]. Nevertheless, this study corroborates existing studies on the importance of identity development and its significance on the mental health of Asian American adolescents.

It should be noted that identity development among Asian American youth might have been even more complicated in recent years because of a spike in anti-Asian racism and sociopolitical tensions in the U.S. [74]. Although we did not include racial discrimination as one of the predictors to focus on cultural orientation, the MLSAAF team has investigated the associations between racial discrimination and cultural orientation as well as several underlying mechanisms in which the experience of racial discrimination differently influences American identity in contrast to ethnic identity. These recent projects are currently under review for publication. The findings from this and those studies as well as from other scholars (e.g., [75]) should collectively provide insights into the nuanced and variant mechanisms in which racial discrimination predicts youth outcomes, either mediated or moderated by cultural orientation.

### 4.3. Ethnic Group Differences

Interaction analyses show that subgroup differences were largely not significant. This is consistent with existing studies that suggest that subgroup differences are likely derived from the different patterns and rates of developmental factors (i.e., higher vs. lower bilingualism or ethnic identity) but not from how each factor shapes youth development differently, i.e., nonsignificant moderation effect. Although in small numbers, significant differences were nevertheless found to suggest that we cannot blindly lump these groups together and should always err on the side of caution before attributing any group differences to a ubiquitous and unspecific “culture”.

In terms of subgroup differences in youth adjustment, it is worth noting that youth behaviors were not statistically and significantly different across Filipino American and Korean American youth in this study. This pattern is in contrast to previous studies that used nationally representative data such as Add Health (e.g., [26]) in which Filipino American youth reported poorer adjustments. The MLSAAF data and Add Health data were collected in different years (2014 and after vs. 1994). Thus, it is plausible that youth behaviors have changed over a 20-year period. It is also possible that the non-significant difference may be because of the sampling strategy. Add Health was nationally representative and used school-based sampling, thus it included a wider group of Filipino Americans. Conversely, the MLSAAF is regional and used a family-based sampling that required parental active consent to interview youth. We learned during the data collection process that Filipino American parents were less likely to participate when their children had difficulties, which was also the case with Korean American parents but to a lesser degree. Thus, it is possible that the MLSAAF samples may not include Filipino American youth who might have had more adjustment difficulties.

### 4.4. Limitations

This study has a few limitations to mention. As indicated in the previous paragraph, the study is regional and cannot be generalized to other Asian American subgroups or Filipino Americans and Korean Americans in different regions. In addition, the measures of internalizing problems could have been more extensive, especially given their seriously high rates among the target groups. However, anxiety was not included in the Wave 1 survey and the rates of suicidality items at Wave 1 were lower than later waves. Due to the lack of variance, they were not included in this study.

## 5. Conclusions

This study helps explain the opportunities and challenges of growing up Asian Americans and its relation to the Asian American youth paradox. The paradox may not be explained by any particular cluster or one predictor. Rather, several traits of Asian American families both in universal predictors and cultural orientation variables complicate youth development, resulting in mixed outcome. In addition, current sociopolitical environments further complicate Asian American youth development. Spikes in sociopolitical tensions and racial hostility in the last decade have contributed to the exacerbation of mental health problems among Asian Americans [74]. Even before the COVID-19 pandemic, racism against Asians was sharply increasing [76,77,78]. The pandemic, however, has provoked an unparalleled intensification in anti-Asian hostility and violence, aggravating distress for Asian Americans [79,80]. To promote positive youth development, healthy and safe sociocultural environments should be provided to assist Asian American families raise their children to develop to their full potential. Unfortunately, those social changes may take time to come. In the meanwhile, Asian American families should be assisted to strengthen their parent-child relations and better manage acculturative differences in the family to reduce the disturbing rate of mental distress and pave the way toward a healthy and positive development.

## Data Availability

The MLSAAF data is available to use. If interested, contact mlsaaf@crownschool.uchicago.edu or visit us at our website, www.mlsaaf.org. There is a procedure in place to submit a proposal for approval.

## Supplementary Materials

The following supporting information can be downloaded at: https://www.mdpi.com/article/10.3390/children11080950/s1, Table S1: Descriptives: Proportions and Mean Differences of Outcome Variables, and Correlations; Table S2: Correlations between Study Variables by Ethnicity; Table S3: Regression Results for Substance Use; Table S4: Regression Results for Antisocial Behaviors; Table S5: Regression Results for GPA; Table S6: Regression Results for Depressive Symptoms; Figure S1: Two-Way Interaction Effect between Peer Antisocial Behavior and Ethnicity on Wave 1 Depressive Symptoms; Figure S2: Two-way Interaction Effect between Heritage Cultural Practice and Ethnicity on Wave 1 Depressive Symptoms.
