# Supplementary material for: A Pathway to Positive Youth Development: Unpacking the Asian American Youth Paradox and Cultural Orientations among Filipino American and Korean American Youth
Source: Children (Basel). 2024 Aug 6;11(8):950. doi: 10.3390/children11080950 (PMC11352463; doi:10.3390/children11080950)
Supplement: Supplementary file 1 [file children-11-00950-s001.zip › children-3076480-supplementary.pdf]

[Table S1] Descriptives: Proportions and Mean Differences of Outcome Variables, and Correlations

Variable Names	All[1]	Substance Use[2]	Antisocial Behavior[3]		GPA[3]		Depression[3]	
	(*N*=786)	(YES=171)	Wave 1	Wave 2	Wave 1	Wave 2	Wave 1	Wave 2
Demographics								
Ethnicity (FA, KA)	48%, 52%	24%, 20%	0.90, 0.77	0.99, 0.75	3.48, 3.54	3.53, 3.52	1.81, 1.81	1.88, 1.97
Nativity (US, foreign born)	64%, 36%	20%, 26%	0.88, 0.81	0.70, 0.94	3.49, 3.54	3.50, 3.56	1.83, 1.77	1.92, 1.95
Gender (male, female)	48%, 52%	22%, 22%	0.96, 0.72*	0.87, 0.86	3.44, 3.57***	3.48, 3.56*	1.73, 1.89**	1.71, 2.12***
Socioeconomic status	3.07 (0.64)	3.02 (0.67)	− 0.09*	− 0.02	− 0.00	0.02	− 0.13***	− 0.11**
Age	15.00 (1.91)	16.38*** (1.51)	0.17***	0.09*	− 0.18***	− 0.16***	0.20***	0.11*
Cultural Orientations								
English	4.71 (0.54)	4.69 (0.59)	− 0.04	− 0.00	0.12***	0.05	− 0.06	− 0.06
Ethnic language	2.97 (1.16)	3.08 (1.20)	− 0.05	− 0.11**	0.08*	0.05	− 0.03	− 0.03
American cultural practice	4.08 (0.73)	4.16 (0.73)	0.05	0.10*	0.01	0.06	− 0.02	− 0.08
Ethnic cultural practice	2.81 (0.83)	2.84 (0.90)	− 0.02	− 0.00	0.05	− 0.04	0.06	0.08*
American identity	3.69 (0.84)	3.58* (0.81)	− 0.03	− 0.01	0.03	− 0.02	− 0.12**	− 0.11**
Ethnic identity	4.25 (0.68)	4.28 (0.65)	− 0.01	− 0.03	− 0.01	0.01	− 0.10**	− 0.04
Universal Factors								
Antisocial belief	1.52 (0.53)	1.97*** (0.58)	0.46***	0.21***	− 0.13***	− 0.13**	0.30***	0.16***
Peer antisocial behaviors	1.31 (0.35)	1.62*** (0.47)	0.46***	0.22***	− 0.22***	− 0.16***	0.16***	0.01
Parent-child Conflict	2.32 (0.86)	2.62*** (0.85)	0.31***	0.18***	− 0.09*	− 0.12**	0.37***	0.21***
Parent-child bonding	3.99 (0.73)	3.83** (0.79)	− 0.24***	− 0.18***	0.10**	0.15***	− 0.34***	− 0.20***
Explicit affection	3.78 (0.79)	3.66 (0.83)	− 0.21***	− 0.13**	0.10**	0.15***	− 0.32***	− 0.23***
Parental rule	3.28 (1.76)	3.09 (1.78)	0.03	− 0.02	− 0.04	0.00	− 0.06	− 0.08
Parental monitor	4.43 (0.70)	4.17*** (0.85)	− 0.26***	− 0.13***	0.16***	0.18***	− 0.02	0.00

*** p<0.001, ** p<0.01, * p<0.05

[1] Percentage of each category of ethnicity, nativity, and gender in the entire sample; average of SES, income, age, and each predicting variable of the entire sample and standard deviation in the parenthesis.

[2] Percentage of those who answered that they had tried any substance in each category of ethnicity, nativity, and gender; average of SES, income, age, and each predicting variable of those who answered that they had tried any substance; asterisk indicating significant difference compared to the group who had not experienced any substance use.

[3] Average of each outcome by groups; correlations between continuous outcomes and explaining variables at each wave

[Table S2] Correlations Between Study Variables by Ethnicity

	1	2	3	4	5	6	7	8	9	10	11	12	13	14	15	16	17	18	19	20	21	22	23	24
1. Substance Use	—	0.38***	0.29***	-0.17***	-0.15**	0.10*	0.12*	-0.12*	-0.02	0.41***	-0.08	0.52***	0.44***	0.20***	-0.14**	-0.07	-0.09	-0.24***	-0.08	0.10*	0.03	0.03	-0.16**	-0.02
2. Antisocial W1	0.34***	—	0.41***	-0.21***	-0.26***	0.16***	0.06	-0.10*	-0.10*	0.19***	-0.15**	0.47***	0.43***	0.31***	-0.27***	-0.20***	0.02	-0.27***	-0.08	-0.06	0.06	-0.08	-0.12*	-0.06
3. Antisocial W2	0.21***	0.36***	—	-0.09	-0.21***	0.12*	0.19***	0.02	-0.05	0.10	-0.14*	0.24***	0.31***	0.21***	-0.14**	-0.08	-0.01	-0.14**	-0.05	-0.05	0.11	-0.00	-0.08	-0.04
4. GPA W1	-0.19***	-0.21***	0.01	—	0.45***	-0.12*	-0.02	-0.03	-0.06	-0.11*	0.15**	-0.12*	-0.21***	-0.04	0.07	0.09	-0.01	0.16**	0.12*	0.09	0.00	0.03	0.13*	0.04
5. GPA W2	-0.14*	-0.18**	-0.03	0.60***	—	-0.08	-0.10	0.02	-0.00	-0.15**	0.10	-0.16**	-0.17**	-0.13*	0.22***	0.22***	-0.01	0.21***	0.04	0.00	0.05	-0.03	0.04	0.04
6. Depression W1	0.09	0.27***	0.14*	-0.01	-0.09	—	0.43***	0.08	-0.09	0.24***	0.04	0.27***	0.22***	0.36***	-0.25***	-0.23***	-0.02	-0.01	-0.06	-0.15**	0.02	-0.03	-0.10*	-0.17***
7. Depression W2	0.07	0.12	0.28***	-0.01	-0.10	0.51***	—	0.02	-0.04	0.10	0.24***	0.14*	0.03	0.23***	-0.11*	-0.16**	-0.07	0.01	-0.02	-0.08	-0.08	0.05	-0.10	-0.04
8. Nativity	-0.03	0.06	0.10	-0.05	-0.18**	-0.02	-0.06	—	-0.05	-0.21***	-0.04	-0.07	-0.02	-0.09	0.05	-0.03	0.15**	0.06	0.28***	-0.41***	0.13**	-0.20***	0.38***	-0.10*
9. SES	-0.07	-0.08	0.01	0.08	0.03	-0.18***	-0.22***	0.06	—	-0.14**	-0.03	-0.11*	-0.06	-0.08	0.20***	0.16**	0.07	0.02	-0.06	0.14**	0.07	0.09	0.06	0.17***
10. Age	0.35***	0.14**	0.07	-0.24***	-0.18**	0.16**	0.13*	-0.07	-0.01	—	0.01	0.38***	0.25***	0.25***	-0.19***	-0.05	-0.19***	-0.09	0.02	0.12*	0.05	0.07	-0.08	-0.05
11. Gender	0.08	-0.03	0.10	0.13*	0.07	0.17**	0.27***	-0.02	-0.04	0.10	—	-0.14**	-0.23***	0.01	0.10*	0.04	-0.03	0.19***	0.14**	0.18***	0.01	0.27***	0.02	0.14**
12. Antisocial beliefs	0.37***	0.44***	0.17**	-0.14**	-0.11	0.32***	0.18**	0.04	-0.04	0.37***	0.01	—	0.48***	0.35***	-0.33***	-0.25***	-0.09	-0.26***	0.03	-0.09	0.11*	-0.09	-0.12*	-0.15**
13. Peer antisocial	0.48***	0.50***	0.14*	-0.22***	-0.15*	0.12*	0.00	-0.01	0.01	0.36***	0.07	0.44***	—	0.20***	-0.14**	-0.08	0.00	-0.26***	-0.07	-0.10*	0.09	-0.03	-0.08	-0.10*
14. Conflict	0.16**	0.31***	0.13*	-0.11*	-0.12	0.39***	0.21***	0.01	-0.05	0.19***	0.09	0.36***	0.27***	—	-0.44***	-0.38***	0.13**	-0.08	0.03	-0.02	0.08	-0.00	-0.05	-0.09
15. Bonding	-0.09	-0.21***	-0.20***	0.12*	0.08	-0.43***	-0.30***	0.03	0.08	-0.09	-0.06	-0.21***	-0.06	-0.48***	—	0.69***	0.09	0.28***	0.06	0.13*	0.13*	0.12*	0.15**	0.27***
16. Explicit affection	-0.08	-0.22***	-0.17**	0.11*	0.08	-0.40***	-0.31***	0.06	0.10	-0.11*	-0.02	-0.24***	-0.13*	-0.48***	0.73***	—	0.02	0.29***	0.09	0.18***	0.18***	0.09	0.16**	0.27***
17. Parental rules	-0.05	0.01	-0.07	-0.05	0.01	-0.10	-0.05	-0.01	-0.03	-0.10	0.07	-0.21***	-0.10	-0.05	0.15**	0.17***	—	0.05	0.14**	-0.14**	0.10*	-0.02	0.15**	0.04
18. Parental monitor	-0.16**	-0.27***	-0.13*	0.18***	0.12*	-0.02	0.01	-0.10*	-0.04	-0.17**	0.15**	-0.28***	-0.26***	-0.22***	0.21***	0.25***	0.14**	—	0.14**	0.08	0.10*	0.09	0.18***	0.16**
19. English	0.06	0.01	0.01	0.20***	0.09	-0.09	-0.10	0.23***	0.11*	0.03	0.16**	0.09	0.04	0.07	0.09	0.10*	-0.08	-0.06	—	-0.20***	0.38***	-0.23***	0.45***	0.03
20. Heritage language	0.05	0.00	-0.11	0.04	0.10	0.07	-0.01	-0.51***	-0.09	0.12*	0.17***	-0.04	0.08	-0.02	0.03	-0.02	0.08	0.08	-0.16**	—	-0.22***	0.49***	-0.22***	0.32***
21. American practice	0.10	0.02	0.04	0.06	0.09	-0.06	-0.06	0.07	0.09	0.12*	0.22***	0.09	0.08	0.09	0.15**	0.17**	0.03	-0.04	0.25***	-0.12*	—	-0.21***	0.44***	0.09
22. Heritage practice	0.02	0.06	0.02	0.05	-0.06	0.16**	0.11	-0.19***	-0.17***	0.00	0.23***	-0.08	-0.01	-0.06	0.07	0.09	0.14**	0.11*	-0.13*	0.50***	-0.17**	—	-0.15**	0.39***
23. American identity	0.01	0.04	0.02	-0.04	-0.10	-0.15**	-0.11	0.40***	0.10*	-0.01	0.01	-0.07	-0.03	-0.05	0.17***	0.24***	0.10*	-0.00	0.27***	-0.30***	0.38***	-0.16**	—	0.13*
24. Ethnic identity	0.05	0.03	-0.05	-0.04	-0.02	-0.03	-0.02	-0.18***	-0.07	0.02	0.20***	-0.08	0.05	-0.01	0.15**	0.12*	0.15**	0.08	-0.03	0.42***	0.01	0.51***	0.06	—

Below the diagonal are correlations for FA youth and above for KA youth.

*** *p* < .001. ** *p* < .01. * *p* < .05.

Antisocial = Antisocial behavior; Nativity (1 = U.S.-born, 0 = Foreign-born); Gender (1 = Female, 0 = Male); Peer antisocial = Peer antisocial behavior; Conflict = Parent-child conflict; Bonding = Parent-child bonding; American practice = American cultural practice; Heritage practice = Heritage cultural practice.

[Table S3] Regression Results for Substance Use

		Wave 1		
Model		Model 1(a)	Model 1(b)	Model 2
Demographics				
Ethnicity	-0.04	0.13	-0.03	0.09
	(0.20)	(0.25)	(0.22)	(0.28)
Nativity (U.S.-Born)	-0.16	-0.28	0.07	-0.06
	(0.20)	(0.23)	(0.25)	(0.29)
SES	-0.04	0.02	-0.05	0.02
	(0.09)	(0.11)	(0.10)	(0.11)
Age	1.09***	0.72***	1.09***	0.73***
	(0.12)	(0.13)	(0.12)	(0.14)
Gender (Female)	-0.16	0.22	-0.19	0.21
	(0.19)	(0.23)	(0.21)	(0.25)
Universal Factors				
Antisocial beliefs		0.69***		0.72***
		(0.12)		(0.12)
Peer antisocial behavior		0.76***		0.76***
		(0.12)		(0.12)
Parent-child conflict		0.02		0.02
		(0.13)		(0.14)
Parent-child bonding		-0.10		-0.13
		(0.16)		(0.16)
Explicit affection		0.15		0.16
		(0.16)		(0.16)
Parental rules		-0.01		-0.02
		(0.12)		(0.13)
parental monitor		-0.13		-0.13
		(0.11)		(0.12)
Cultural Orientations				
English			-0.07	-0.17
			(0.11)	(0.13)
Heritage language			0.02	0.01
			(0.13)	(0.16)

American cultural practice			0.23+	0.02
			(0.12)	(0.14)
Heritage cultural practice			-0.02	0.05
			(0.13)	(0.15)
American identity			-0.27*	-0.03
			(0.12)	(0.15)
Ethnic identity			0.08	0.16
			(0.12)	(0.14)
Observations	748	735	746	733
R-squared	0.149	0.326	0.160	0.333

*** $p<0.001$, ** $p<0.01$, * $p<0.05$, + $p<0.1$

R^2 for categorical outcomes are adjusted R^2 (pseudo R^2).

[Table S4] Regression Results for Antisocial Behavior

Wave	Wave 1				Wave 2				Wave 2 w/ Wave 1			
Model		Model 1(a)	Model 1(b)	Model 2		Model 1(a)	Model 1(b)	Model 2		Model 1(a)	Model 1(b)	Model 2
Demographics												
Ethnicity	-0.18	-0.00	-0.15	0.04	-0.22	-0.13	-0.20	-0.07	-0.17	-0.14	-0.19	-0.12
	(0.12)	(0.11)	(0.14)	(0.12)	(0.16)	(0.16)	(0.17)	(0.18)	(0.15)	(0.16)	(0.16)	(0.17)
Nativity (U.S.-Born)	-0.03	-0.14	-0.01	-0.16	0.28+	0.25	0.31+	0.25	0.26+	0.27+	0.30+	0.28
	(0.13)	(0.11)	(0.15)	(0.13)	(0.16)	(0.16)	(0.19)	(0.19)	(0.15)	(0.16)	(0.18)	(0.18)
SES	-0.13*	-0.06	-0.14*	-0.06	-0.04	0.04	0.00	0.06	0.01	0.05	0.03	0.07
	(0.06)	(0.05)	(0.06)	(0.05)	(0.08)	(0.08)	(0.08)	(0.08)	(0.07)	(0.08)	(0.07)	(0.08)
Age	0.30***	-0.05	0.31***	-0.03	0.18*	-0.07	0.16+	-0.08	0.07	-0.05	0.04	-0.06
	(0.06)	(0.06)	(0.06)	(0.06)	(0.08)	(0.09)	(0.08)	(0.09)	(0.08)	(0.09)	(0.08)	(0.09)
Gender (Female)	-0.36**	-0.14	-0.32*	-0.11	-0.10	0.07	-0.10	0.00	0.05	0.12	0.03	0.04
	(0.12)	(0.11)	(0.13)	(0.11)	(0.15)	(0.15)	(0.16)	(0.16)	(0.14)	(0.15)	(0.15)	(0.16)
W1 outcome									0.36***	0.27***	0.35***	0.28***
									(0.05)	(0.06)	(0.05)	(0.06)
Universal Factors												
Antisocial beliefs		0.32***		0.33***		0.13		0.10		0.02		-0.00
		(0.06)		(0.06)		(0.09)		(0.09)		(0.09)		(0.09)
Peer antisocial behavior		0.35***		0.35***		0.28**		0.28**		0.17*		0.15+
		(0.05)		(0.05)		(0.09)		(0.08)		(0.09)		(0.09)
Parent-child conflict		0.21***		0.20**		0.19*		0.15+		0.11		0.09
		(0.06)		(0.06)		(0.09)		(0.09)		(0.09)		(0.09)
Parent-child bonding		-0.16*		-0.19**		-0.23*		-0.27*		-0.15		-0.18
		(0.07)		(0.07)		(0.11)		(0.11)		(0.11)		(0.11)
Explicit affection		0.01		0.02		0.03		0.02		-0.03		-0.04
		(0.07)		(0.07)		(0.10)		(0.10)		(0.10)		(0.10)
Parental rules		0.11+		0.10+		-0.06		-0.05		-0.06		-0.06
		(0.06)		(0.06)		(0.08)		(0.08)		(0.08)		(0.08)
parental monitor		-0.14**		-0.14**		-0.06		-0.06		-0.04		-0.03
		(0.05)		(0.05)		(0.08)		(0.08)		(0.08)		(0.08)
Cultural Orientations												
English			-0.13+	-0.15**			-0.11	-0.05			-0.08	-0.04
			(0.07)	(0.06)			(0.09)	(0.09)			(0.08)	(0.09)
Heritage language			-0.11	-0.15*			-0.16	-0.19+			-0.10	-0.13

			(0.08)	(0.07)			(0.11)	(0.11)			(0.10)	(0.11)
American cultural practice			0.14+	0.00			0.25**	0.19*			0.22*	0.21*
			(0.07)	(0.06)			(0.09)	(0.09)			(0.09)	(0.09)
Heritage cultural practice			0.01	0.09			0.17	0.25*			0.17+	0.22*
			(0.08)	(0.07)			(0.10)	(0.10)			(0.10)	(0.10)
American identity			-0.09	0.06			-0.17+	-0.09			-0.15	-0.11
			(0.08)	(0.07)			(0.10)	(0.10)			(0.09)	(0.10)
Ethnic identity			0.03	0.06			-0.07	-0.01			-0.09	-0.05
			(0.07)	(0.06)			(0.09)	(0.09)			(0.08)	(0.09)
Observations	757	744	755	742	580	570	578	568	578	568	576	566
R-squared	0.0196	0.131	0.0250	0.138	0.00789	0.0379	0.0177	0.0456	0.0455	0.0553	0.0532	0.0622

*** $p<0.001$, ** $p<0.01$, * $p<0.05$, + $p<0.1$

R^2 for categorical outcomes are adjusted R^2 (pseudo R^2)

[Table S5] Regression Results for GPA

Wave	Wave 1				Wave 2				Wave 2 w/ Wave 1			
Model		Model 1(a)	Model 1(b)	Model 2		Model 1(a)	Model 1(b)	Model 2		Model 1(a)	Model 1(b)	Model 2
Demographics												
Ethnicity	0.04	0.01	0.02	-0.01	-0.03	-0.06	-0.02	-0.05	-0.05	-0.06	-0.02	-0.04
	(0.04)	(0.04)	(0.04)	(0.04)	(0.04)	(0.04)	(0.05)	(0.05)	(0.04)	(0.04)	(0.04)	(0.04)
Nativity (U.S.-Born)	-0.07+	-0.05	-0.11*	-0.09+	-0.10*	-0.09*	-0.09+	-0.08	-0.05	-0.05	-0.02	-0.02
	(0.04)	(0.04)	(0.05)	(0.05)	(0.04)	(0.04)	(0.05)	(0.05)	(0.04)	(0.04)	(0.04)	(0.04)
SES	-0.01	-0.01	-0.01	-0.01	0.00	-0.00	-0.00	-0.01	0.01	0.01	0.01	0.01
	(0.02)	(0.02)	(0.02)	(0.02)	(0.02)	(0.02)	(0.02)	(0.02)	(0.02)	(0.02)	(0.02)	(0.02)
Age	-0.10***	-0.07***	-0.11***	-0.08***	-0.09***	-0.07**	-0.10***	-0.07**	-0.05*	-0.04*	-0.05*	-0.04+
	(0.02)	(0.02)	(0.02)	(0.02)	(0.02)	(0.02)	(0.02)	(0.02)	(0.02)	(0.02)	(0.02)	(0.02)
Gender (Female)	0.14***	0.11**	0.11**	0.09*	0.09*	0.07+	0.09*	0.08+	0.03	0.03	0.04	0.05
	(0.04)	(0.04)	(0.04)	(0.04)	(0.04)	(0.04)	(0.04)	(0.04)	(0.04)	(0.04)	(0.04)	(0.04)
W1 outcome									0.49***	0.48***	0.50***	0.49***
									(0.04)	(0.04)	(0.04)	(0.04)
Universal Factors												
Antisocial beliefs		0.01		0.00		0.01		-0.00		0.01		-0.00
		(0.02)		(0.02)		(0.02)		(0.02)		(0.02)		(0.02)
Peer antisocial behavior		-0.08***		-0.07***		-0.05*		-0.05*		-0.00		-0.00
		(0.02)		(0.02)		(0.02)		(0.02)		(0.02)		(0.02)
Parent-child conflict		0.01		0.00		-0.00		-0.01		-0.01		-0.02
		(0.02)		(0.02)		(0.02)		(0.02)		(0.02)		(0.02)
Parent-child bonding		-0.00		0.00		0.04		0.04		0.03		0.02
		(0.03)		(0.03)		(0.03)		(0.03)		(0.03)		(0.03)
Explicit affection		0.03		0.03		0.03		0.03		0.02		0.02
		(0.03)		(0.03)		(0.03)		(0.03)		(0.03)		(0.03)
Parental rules		-0.04*		-0.04+		-0.03		-0.02		-0.02		-0.01
		(0.02)		(0.02)		(0.02)		(0.02)		(0.02)		(0.02)
parental monitor		0.05*		0.04*		0.05*		0.05*		0.02		0.02
		(0.02)		(0.02)		(0.02)		(0.02)		(0.02)		(0.02)
Cultural Orientations												
English			0.09***	0.08***			0.03	0.02			-0.02	-0.02
			(0.02)	(0.02)			(0.02)	(0.02)			(0.02)	(0.02)
Heritage language			0.05+	0.05+			0.04	0.04			0.01	0.00

			(0.03)	(0.03)			(0.03)	(0.03)			(0.02)	(0.02)
American cultural practice			-0.01	-0.01			0.04	0.04+			0.05*	0.05*
			(0.02)	(0.02)			(0.02)	(0.02)			(0.02)	(0.02)
Heritage cultural practice			0.02	0.01			-0.04+	-0.05+			-0.05*	-0.06*
			(0.02)	(0.02)			(0.03)	(0.03)			(0.02)	(0.02)
American identity			0.03	0.02			-0.02	-0.03			-0.04+	-0.05*
			(0.02)	(0.02)			(0.02)	(0.03)			(0.02)	(0.02)
Ethnic identity			-0.05*	-0.04*			-0.01	-0.01			0.02	0.01
			(0.02)	(0.02)			(0.02)	(0.02)			(0.02)	(0.02)
Observations	734	722	732	720	567	558	565	556	549	540	547	538
R-squared	0.06	0.10	0.09	0.13	0.04	0.08	0.06	0.10	0.30	0.31	0.31	0.33

*** $p<0.001$, ** $p<0.01$, * $p<0.05$, + $p<0.1$

[Table S6] Regression Results for Depressive Symptoms

Wave	Wave 1				Wave 2				Wave 2 w/ Wave 1			
Model		Model 1(a)	Model 1(b)	Model 2		Model 1(a)	Model 1(b)	Model 2		Model 1(a)	Model 1(b)	Model 2
Demographics												
Ethnicity	0.05	0.10+	-0.01	0.05	0.14*	0.16*	0.14+	0.17*	0.14*	0.12+	0.16*	0.15*
	(0.05)	(0.05)	(0.06)	(0.06)	(0.07)	(0.07)	(0.08)	(0.08)	(0.06)	(0.07)	(0.07)	(0.07)
Nativity (U.S.-Born)	0.13*	0.14**	0.20**	0.20**	0.01	0.03	0.02	0.03	-0.03	-0.01	-0.08	-0.06
	(0.06)	(0.05)	(0.07)	(0.06)	(0.07)	(0.07)	(0.08)	(0.08)	(0.06)	(0.07)	(0.08)	(0.08)
SES	-0.08**	-0.04+	-0.07*	-0.04	-0.08*	-0.04	-0.05	-0.03	-0.02	-0.01	-0.01	-0.00
	(0.03)	(0.02)	(0.03)	(0.02)	(0.03)	(0.03)	(0.03)	(0.03)	(0.03)	(0.03)	(0.03)	(0.03)
Age	0.14***	0.06*	0.15***	0.07*	0.07+	0.01	0.08*	0.02	-0.00	-0.02	0.01	-0.01
	(0.03)	(0.03)	(0.03)	(0.03)	(0.04)	(0.04)	(0.04)	(0.04)	(0.03)	(0.04)	(0.03)	(0.04)
Gender (Female)	0.14**	0.14**	0.16**	0.14**	0.40***	0.40***	0.44***	0.43***	0.35***	0.34***	0.38***	0.36***
	(0.05)	(0.05)	(0.06)	(0.05)	(0.07)	(0.07)	(0.07)	(0.07)	(0.06)	(0.06)	(0.06)	(0.07)
W1 outcome									0.51***	0.47***	0.50***	0.46***
									(0.04)	(0.05)	(0.04)	(0.05)
Universal Factors												
Antisocial beliefs		0.10***		0.10***		0.09*		0.09*		0.04		0.04
		(0.03)		(0.03)		(0.04)		(0.04)		(0.04)		(0.04)
Peer antisocial behavior		0.03		0.03		-0.02		-0.02		-0.05		-0.04
		(0.03)		(0.03)		(0.04)		(0.04)		(0.04)		(0.04)
Parent-child conflict		0.15***		0.17***		0.09*		0.11**		0.02		0.04
		(0.03)		(0.03)		(0.04)		(0.04)		(0.04)		(0.04)
Parent-child bonding		-0.11**		-0.10**		-0.04		-0.03		-0.00		-0.00
		(0.04)		(0.04)		(0.05)		(0.05)		(0.05)		(0.05)
Explicit affection		-0.07+		-0.05		-0.11*		-0.09+		-0.07		-0.06
		(0.04)		(0.04)		(0.05)		(0.05)		(0.04)		(0.05)
Parental rules		-0.02		-0.02		-0.04		-0.03		-0.03		-0.03
		(0.03)		(0.03)		(0.04)		(0.04)		(0.03)		(0.03)
parental monitor		0.08**		0.09**		0.05		0.06		0.01		0.02
		(0.03)		(0.03)		(0.04)		(0.04)		(0.03)		(0.03)
Cultural Orientations												
English			-0.07*	-0.08**			-0.06	-0.06			-0.01	-0.01
			(0.03)	(0.03)			(0.04)	(0.04)			(0.04)	(0.04)

Heritage language			-0.06	-0.05			-0.13**	-0.12**			-0.10*	-0.10*
			(0.04)	(0.03)			(0.05)	(0.05)			(0.04)	(0.04)
American cultural practice			0.05	0.03			-0.04	-0.04			-0.07+	-0.05
			(0.03)	(0.03)			(0.04)	(0.04)			(0.04)	(0.04)
Heritage cultural practice			0.09*	0.10**			0.06	0.07+			0.02	0.04
			(0.03)	(0.03)			(0.04)	(0.04)			(0.04)	(0.04)
American identity			-0.11***	-0.06+			-0.07+	-0.03			-0.01	-0.00
			(0.03)	(0.03)			(0.04)	(0.04)			(0.04)	(0.04)
Ethnic identity			-0.09**	-0.06*			-0.04	-0.02			-0.00	-0.00
			(0.03)	(0.03)			(0.04)	(0.04)			(0.04)	(0.04)
Observations	749	736	747	734	580	570	578	568	573	563	571	561
R-squared	0.06	0.24	0.11	0.27	0.08	0.15	0.11	0.18	0.27	0.28	0.28	0.30

*** p<0.001, ** p<0.01, * p<0.05, + p<0.1

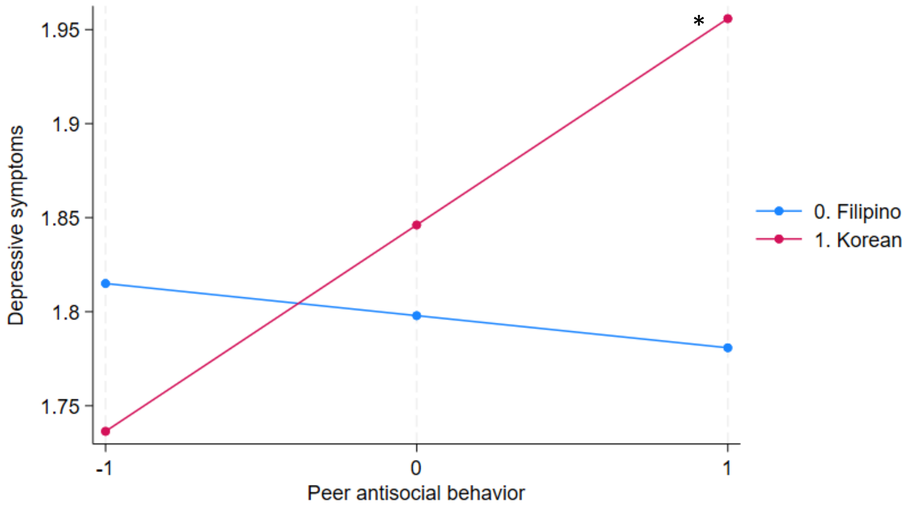

Figure S1 Two-Way Interaction Effect Between Peer Antisocial Behavior and Ethnicity on Wave 1 Depressive Symptoms. *Note:* Asterisk (*) indicates the significance of the slope (p < .05).

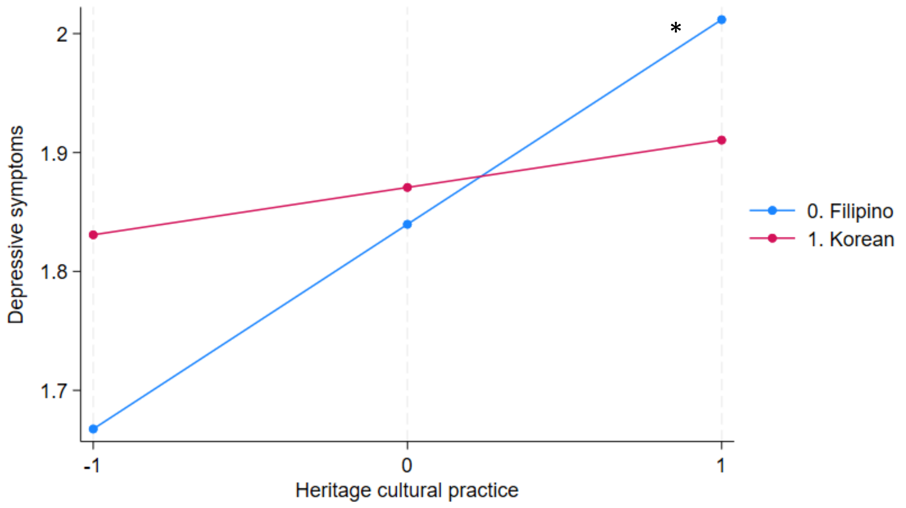

Figure S2. Two-Way Interaction Effect Between Heritage Cultural Practice and Ethnicity on Wave 1 Depressive Symptoms. *Note*: Asterisk (*) indicates the significance of the slope ($p < .05$).
